# Exercise, type 1 diabetes mellitus and blood glucose: The implications of exercise timing

**DOI:** 10.3389/fendo.2022.1021800

**Published:** 2022-09-28

**Authors:** Ross Fitzpatrick, Gareth Davison, Jason J. Wilson, Gerard McMahon, Conor McClean

**Affiliations:** Sport and Exercise Sciences Research Institute, Ulster University, Newtownabbey, Ireland

**Keywords:** exercise, circadian, glucose metabolism, type 1 diabetes mellitus, molecular clock

## Abstract

The scientific literature shows that exercise has many benefits for individuals with type 1 diabetes. Yet, several barriers to exercise in this population exist, such as post-exercise hypoglycaemia or hyperglycaemia. Several studies suggest that the timing of exercise may be an important factor in preventing exercise-induced hypoglycaemia or hyperglycaemia. However, there is a paucity of evidence solely focused on summarising findings regarding exercise timing and the impact it has on glucose metabolism in type 1 diabetes. This report suggests that resistance or high-intensity interval exercise/training (often known as HIIT) may be best commenced at the time of day when an individual is most likely to experience a hypoglycaemic event (i.e., afternoon/evening) due to the superior blood glucose stability resistance and HIIT exercise provides. Continuous aerobic-based exercise is advised to be performed in the morning due to circadian elevations in blood glucose at this time, thereby providing added protection against a hypoglycaemic episode. Ultimately, the evidence concerning exercise timing and glycaemic control remains at an embryonic stage. Carefully designed investigations of this nexus are required, which could be harnessed to determine the most effective, and possibly safest, time to exercise for those with type 1 diabetes.

## Introduction

In individuals with type 1 diabetes, exercise is advised for condition management, for general health and well-being, and for reducing the risk of several chronic conditions (1). Nevertheless, many individuals with type 1 diabetes are unable to meet physical activity guidelines, with one recent study reporting that 49% of volunteers do not achieve published recommendations (2). Numerous factors explain this trend, such as lack of time and/or social support, as well as specific type 1 diabetes associated risks with exercise, including exercise-induced hypoglycaemia or hyperglycaemia (1). Hypoglycaemia, for example, can be a risk for several hours following exercise and is a well-recognised barrier to exercise in this cohort (3). Improving personal knowledge of insulin pharmacokinetics may be one approach to help mitigate this barrier (3). For example, reducing insulin basal and/or bolus doses prior to exercise can prevent hypoglycaemia during aerobic exercise, however aggressive reductions can cause hyperglycaemia (4). Another key factor in mitigating this barrier is through developing an understanding of coherent exercise strategies to reduce the risk of hypoglycaemia (3). A certain strategy may involve the consideration of exercise timing to augment the functioning of the biological clock, which is a key regulator of glucose homeostasis (5). For instance, skeletal muscle insulin sensitivity displays circadian rhythmicity that could arguably have implications for insulin administration/timing on blood glucose management around scheduled exercise (6–8). Various studies have thus sought to ascertain an optimal time of day to exercise for glucose management in type 1 diabetes (9–12). In this short *Perspectives* report, we aim to summarise findings in this important domain and identify practical approaches, based on observed circadian responses, to combat barriers to exercise in those with type 1 diabetes.

## Circadian rhythms and type 1 diabetes

The term “*circadian*” is a Latin derivative of the phrase “*Circa Diem*” and translates as “*about a day*”, referring to the ~24-hr diurnal cycle. Almost every cell in the human body exhibits circadian rhythmicity, which is regulated by the molecular clock mechanism (13). The molecular clock controls metabolic, physiological, and behavioural oscillations throughout the 24-hr period, *via* an autoregulatory transcriptional-translational feedback loop (13). People with type 1 diabetes have disturbed molecular clocks, which are partly responsible for increased mortality rates in those with the condition (14). For example, normal blood pressure exhibits a circadian rhythm, dipping during sleep, which is important for cardiovascular health (15). However, individuals with type 1 diabetes have an increased prevalence of non-dipping blood pressure, thereby increasing the risks of hypertension (16) and nephropathy (17). As a result, these complications ultimately contribute to the development of cardiovascular disease as a leading cause of mortality in type 1 diabetes (18). Moreover, social jetlag, a prominent circadian misalignment, is independently associated with long-term blood glucose levels (glycosylated haemoglobin - HbA_1c_) (19), and this may, to some extent, underpin observations that those with type 1 diabetes engaging in shift work (defined as work scheduled outside standard daytime hours and night work of at least 3-hours duration between ~23:00–06:00) present with higher HbA_1c_ compared to those that do not follow shift patterns (20). Appropriate timing of exercise has been postulated to reset the molecular clock and circadian rhythms (21) and, hence, arguably has the potential to improve cardiometabolic health in type 1 diabetes by restoring crucial biological and physiological processes beyond that of a less carefully selected exercise bout.

## Discussion

### Exercise timing in type 1 diabetes

**Figure 1:**
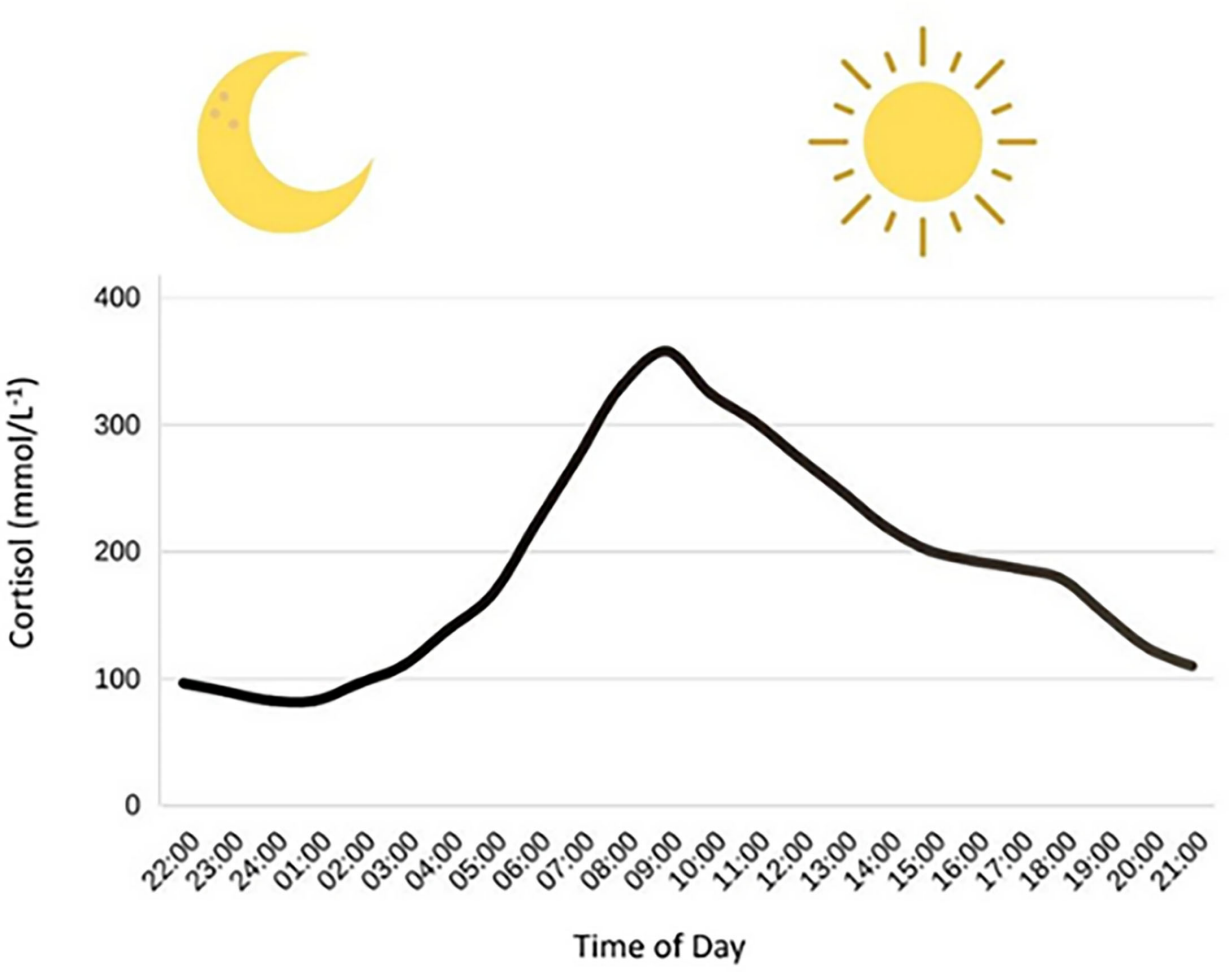
Circadian variation in cortisol concentrations. Cortisol has a nadir around (01:00) and then has a steep rise in the morning hours, eventually hitting a peak at around (09:00). After this peak, cortisol concentrations decline throughout the rest of the day. Figure adapted from Debono et al. (23).

At present, the data suggests that fasted morning exercise triggers a rise in blood glucose following exercise, when compared to postprandial afternoon exercise (9–12). In contrast, recent studies show a decline in glucose following afternoon exercise (10, 11), which may explain the higher rates of hypoglycaemia (10.7 events per individual) following afternoon exercise compared to morning exercise (5.6 events per individual) (9). Mechanistically, morning exercise (~07:00-08:00) may increase blood glucose *via* circadian-mediated elevations in cortisol (**Figure 1**) and growth hormone as part of the ‘Dawn Phenomenon’ (22); this may have a glucose-sparing effect by stimulating lipolysis. However, in the afternoon (~16:00-17:00), growth hormone and cortisol decline, thereby increasing the risk of hypoglycaemia, as gluconeogenesis and glucagon concentration decrease, respectively (9).

**Figure 1**. Circadian variation in cortisol concentrations. Cortisol has a nadir around (01:00) and then has a steep rise in the morning hours, eventually hitting a peak at around (09:00). After this peak, cortisol concentrations decline throughout the rest of the day. Figure adapted from Debono et al.s (23).

Unlike the observations regarding exercise-induced hypoglycaemia in the afternoon (9–11), evidence for the time-of-day effects of exercise-induced hyperglycaemia appears equivocal. Two studies have reported no difference in the number of hyperglycaemic events between exercise timing conditions (9, 10). In contrast, Toghi-Eshghi and Yardley found more hyperglycaemic episodes (12 versus 5) six hours post-exercise in the morning relative to the afternoon (11). Similarly, Ruegemer et al. observed a hyperglycaemic response to morning exercise but not afternoon exercise among participants; the morning hyperglycaemia was mild and short-lived, with minimal impact on overall blood glucose (12). These heterogeneous findings may be explained by differences in exercise intensity and exercise modality. With respect to the latter, a meta-analysis by Garcia-Garcia et al. (24) showed that rapid decays of blood glucose were found during continuous moderate intensity exercise, whereas resistance exercise was associated with more constrained decreases. Aerobic exercise in type 1 diabetes relies heavily on blood glucose as a fuel source, whereas blood glucose during resistance exercise is better protected given that intramuscular glycogen is the primary fuel used (1). Thus, resistance exercise has been shown to provide greater blood glucose stability when compared to aerobic exercise (24). Specifically, Yardley et al. (25) observed a decrease in plasma glucose from 8.4 ± 2.7 to 6.8 ± 2.3 mmol∙L^-1^ during resistance exercise (45-minutes long, consisting of 7 exercises, performing 3 sets of 8 repetitions maximum with 90-seconds rest between sets) compared to 9.2 ± 3.4 to 5.8 ± 2.0 mmol∙L^-1^ during aerobic exercise (45-minutes at 60% of V̇O_2max_) in type 1 diabetes. All exercise sessions in this study were performed at 16:00 and in the fed state (25). Therefore, it is conceivable that morning aerobic exercise results in lower elevations in peripheral blood glucose (12) compared to morning resistance exercise, which appears to intensify the circadian mediated rise in peripheral blood glucose (11), at least when the exercise is performed in a fasted state. An increase in blood glucose during resistance exercise in type 1 diabetes seems to occur only when performed in a fasted state (11, 26–28). A meta-analysis investigating metabolic responses to fed and fasted exercise found that fasted exercise was associated with higher levels of free fatty acids than fed exercise (29). Free fatty acids can induce acute and chronic insulin resistance (30), which may, coupled with the Dawn Phenomenon, underpin the increased blood glucose during fasted exercise, and consequently, the observed post-exercise hyperglycaemia in the fasted morning exercise groups in the studies by Toghi-Eshghi and Yardley (11) and Ruegemer et al. (12). However, further direct studies are warranted to explore the mechanistic basis of such observations in those with type 1 diabetes.

Exercise intensity is also an important consideration in type 1 diabetes, with high-intensity interval exercise and training (HIIT) proving safer than continuous exercise at reducing the risk of hypoglycaemia (31). This may, in part, be due to HIIT causing similar physiological glucose responses as resistance training, again due to greater reliance on intramuscular glycogen and phosphagens over blood glucose as energy (1). Interestingly, Yardley (10) used a HIIT protocol (10-second sprints every 2 minutes for 24 minutes), yet, observed no differences between exercise timing conditions for hyperglycaemic events. Mechanistically, this exercise protocol might be expected to increase hyperglycaemic episodes in the morning, similar to the observations following the resistance exercise used by Toghi-Eshghi and Yardley (11). However, a key difference between studies is that Yardley (10) notably included a 10-minute warm-up and an 11-minute cooldown at 50% V̇O₂_peak_, possibly explaining the reductions in hyperglycaemic effects observed in the morning exercise group compared to Toghi-Eshghi and Yardley (11), who did not include a cooldown protocol. A low-intensity aerobic exercise cool-down may counteract post-exercise hyperglycaemia, however, current evidence for this approach is limited. Mechanistically a cooldown may counteract post-exercise hyperglycaemia *via* increased glucose (32) and lactate oxidation. Lactate is a substrate for gluconeogenesis and without a cooldown, may instead be converted to glucose in the liver resulting in subsequent hyperglycaemia (33, 34). One study has shown that performing running exercise (at 60% V̇o_2peak_), albeit for 45minutes, after a resistance bout improves glycaemic stability throughout exercise and reduces the duration and severity of post-exercise hypoglycaemia (35). Consequently, future research should seek to investigate the integration and effectiveness of realistic cool-down strategies, taking into consideration exercise duration and intensity.

### Study designs and approaches

A point worth mentioning at this juncture is that the sample sizes used among studies are relatively consistent, with most having a sample of between 6-12 participants, apart from Gomez et al. (9) who recruited 32 participants. This increases the weight of the findings by Gomez et al. (9) as small samples are known to disrupt the effect of statistical findings compared to larger samples. Although, only Yardley (10) used a power calculation in which they calculated a sample of 12 participants with an 0.86 power and an alpha of 0.05. Also, the consistent exercise times implemented across studies (07:00 versus 16:00/17:00) allow for valid comparisons between studies. Finally, randomised controlled crossover study designs were ubiquitously used, which are considered the gold standard due to participants acting as their own control, thereby reducing risks of interindividual confounding factors.

### Exercise timing, diet and sleep

In addition to the direct effects of exercise on blood glucose, there may also be indirect effects incurred through the impact exercise has on factors such as diet and sleep. For instance, exercise is known to cause changes in the regulation of appetite, with an acute exercise bout showing a reduction in appetite in both lean and obese individuals (36), possibly through suppression of the hunger hormone ghrelin and increases in satiety hormones peptide YY (PYY), pancreatic polypeptide (PP), and glucagon-like peptide 1 (GLP-1) (37). Gut hormones related to hunger and satiety are known to display diurnal oscillations, with ghrelin displaying peaks in the evening and showing troughs in the morning (38). Considering that humans consume the majority of their daily calories in the evening, and also happen to eat more types of sweet and refined sugar foods in the latter part of the day (39), it is reasonable to theorise that performing an exercise bout (with appetite reducing effects) at this time may reduce caloric intake and improve dietary quality. Therefore, improving dietary intake and/or quality could lead to improved blood glucose in type 1 diabetes, as was shown by Nansel, Lipsky, and Liu (40). Furthermore, an evening exercise bout with its insulin sensitising effects may improve insulin sensitivity at a time in which it is typically worsened (41). Increased insulin sensitivity is an important therapeutic target to reduce the risk of macro- (42) and microvascular (43) complications in type 1 diabetes.

The timing of exercise may additionally impact sleep quality. Sleep quality is an important consideration given that one night of partial sleep deprivation in type 1 diabetes has been shown to cause peripheral insulin resistance (44). The literature investigating the effects of exercise timing on sleep quality demonstrates contrasting findings: Alley et al. found that the timing of resistance exercise had no impact on sleep outcomes (45), and this was supported by Burgess et al. (46) who found that regardless of the time of day exercise is performed, it does not impact sleep quality. Opposingly, Yamanaka et al. (47) found that morning and evening exercise, respectively, differentially impact body temperature and cardiac activity during sleep and stated that morning aerobic exercise may improve sleep quality relative to an identical exercise stimulus performed in the evening, due to allowing time for the exercise-induced stimulation of the sympathetic nervous system to diminish. However, whether these variations manifest in perturbations in health remain unknown as the study failed to investigate any health markers. Furthermore, a systematic review and meta-analysis found no evidence that evening exercise impacts sleep, except for vigorous exercise performed ≤ 1 h before bedtime (48). Therefore, individuals should also consider whether evening exercise impacts their sleep, and perhaps modify exercise time accordingly. It therefore seems likely that performing exercise at any time of day (with the exception being HIIT ≤ 1 h before bedtime) improves sleep outcomes when compared to no exercise at all in those with type 1 diabetes (49), and thus identifies another mechanism through exercise can elicit desirable effects for those with type 1 diabetes.

## Practical approaches

Exercise in type 1 diabetes is clinically endorsed for various reasons, most notably to improve cardiometabolic health (18). Physical activity recommendations from the American Diabetes Association (ADA) include ≥150 minutes of moderate physical activity and ≥2 resistance exercise sessions per week (50). Partaking in resistance or HIIT exercise, instead of continuous exercise, at the time of day an individual is most likely to experience a hypoglycaemic event (i.e., afternoon/evening) may be more prudent and advised because of the superior blood glucose stability these exercise modalities provide (24, 25, 31). Contrastingly, if continuous exercise is the preferred mode of exercise, it may be best commenced in the morning due to known elevations in type 1 diabetes blood glucose concentration at this time, potentially providing added protection against a hypoglycaemic episode during this heavily glucose reliant exercise form (24, 25). Additionally, individuals with type 1 diabetes who regularly exercise are advised to strategically consume carbohydrates before, during and after exercise to protect against a hypoglycaemic episode. The quantity of carbohydrates to be taken depends on factors such as the exercise undertaken (considering mode, duration, and intensity etc.), and present blood glucose/insulin concentrations. For example, when insulin levels are low, it is recommended that an individual should consume 30-60g of carbohydrate per hour to prevent hypoglycaemia during exercise. Whereas, up to 75g of carbohydrate per hour is advised under high insulin conditions (1). Reductions in insulin basal and/or bolus dose can be used in addition to, or alongside, carbohydrate intake for the prevention of exercise-induced hypoglycaemia. Various insulin adjustment strategies can be used, one of which involves reducing the pre-exercise bolus by 30-50% up to 90 minutes before aerobic exercise (51) – decisions around such approaches should be made in consultation with the designated clinical/endocrinology teams and informed by a patient’s/individual’s past experiences and response to exercise. Aerobic exercise may require greater reductions in insulin doses and/or more carbohydrate intake than a HIIT session, whereas resistance training may require increased insulin in the post-exercise recovery phase (1). The order of resistance and aerobic exercise is also an important consideration if both/multiple exercise modalities are completed in the same session. Performing resistance exercise prior to aerobic exercise can keep blood glucose concentration buoyant (35), possibly due to an increase in counter-regulatory hormones that mitigate glucose clearance. This approach can reduce the possibility of a hypoglycaemic episode and may therefore be a practical approach for those individuals who suffer from hypoglycaemia as a function of exercise. For further practical advice on how to manage exercise with type 1 diabetes, we recommend the excellent consensus statement by Riddell et al. (1).

## Future directions

**Figure 2:**
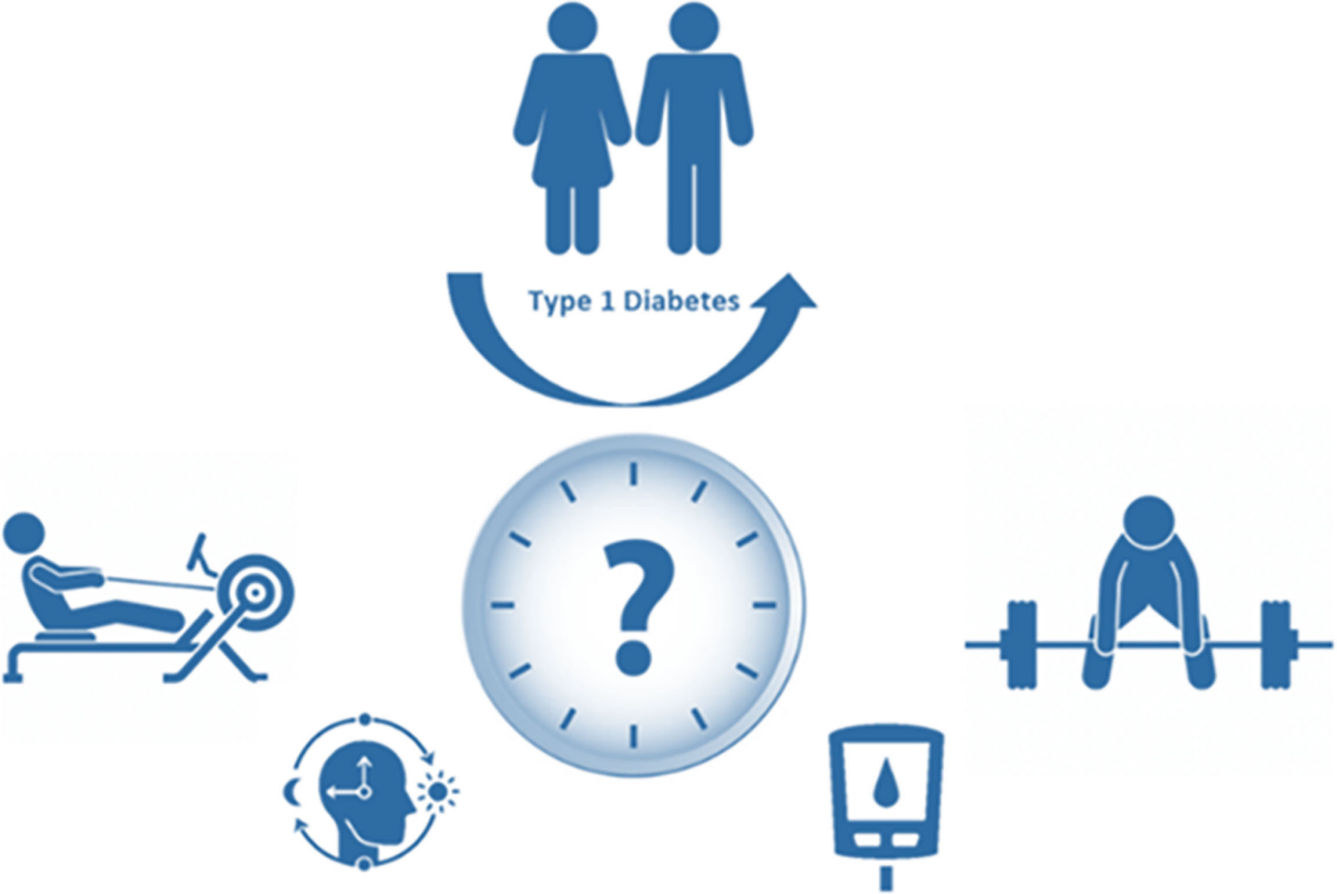
Future research in type 1 diabetes should aim to investigate the impact of exercise timing on different exercise modalities in relation to biological factors such as chronotype. Core blood glucose monitoring (CGM) is a metabolic predictor of specific interest.

All the experimental studies outlined within compared fasted morning exercise with postprandial afternoon exercise. Consequently, experiments were not only investigating the timing of exercise and possible circadian effects, but also implicitly shedding light on the timing of exercise in relation to the fasted and the fed/postprandial state that ultimately requires further scrutiny. This brief synopsis considers peripheral glucose concentration mainly in relation to exercise timing, which is why studies that compared blood glucose responses at the same time of day in a fasted or fed state, such as Yamanouchi et al. (52), were not considered for commentary. We acknowledge that glucose management in this clinical population is multifactorial and inherently complex with exercise regimes requiring careful, individualised tailoring with respect to factors such as carbohydrate intake, insulin adjustments and other personal circumstances. Further research in type 1 diabetes is warranted, including long-term studies exploring the impact of exercise type (resistance vs. aerobic; continuous vs HIIT) and exercise timing (based on personal preference, or even chronotype) on key metabolic and endocrine predictors for those with type 1 diabetes (**Figure 2**). Of particular interest is research on HIIT exercise in relation to exercise timing, as HIIT exercise has been shown to provide similar physiological adaptations as continuous training, while simultaneously reducing the extent of glycogen breakdown, thus providing additional protection against exercise-induced hypoglycaemia (1, 31). Personalised exercise prescription and support may likely hold the key to sustaining adherence and yielding the fullest health benefits for those with type 1 diabetes. Indeed, Lascar et al. (53), highlighted that one-to-one advice from a health and fitness advisor had large appeal to individuals with type 1 diabetes, primarily due to the perception that one-to-one advice would be tailored to individual needs.

## Conclusion

There is increasing scientific interest in how circadian rhythms and molecular clocks interact with a plethora of conditions, such as type 1 diabetes. Disrupted circadian rhythms may contribute to poorer long-term blood glucose management (i.e., increased HbA_1c_) and increased cardiovascular risk in type 1 diabetes. Due to this interaction, clinicians and other professionals who are interested and involved in those with type 1 diabetes may consider factors such as exercise timing to maximise the therapeutic outcomes of an exercise bout. Furthermore, prescribing a suitable time to exercise may reduce the occurrence of a hypoglycaemic event, and thus assist in removing a common perceived barrier to exercise in type 1 diabetes. We state that the benefits of exercise in type 1 diabetes far outweigh the inherent risks, but also remain cognisant of the numerous challenges in trying to safely incorporate consistent activity into a daily schedule: carefully choosing the time of day to exercise might be one such approach to facilitate a more active lifestyle in type 1 diabetes.

## Data availability statement

The original contributions presented in the study are included in the article/supplementary material. Further inquiries can be directed to the corresponding author.

## Author contributions

J W, RF, and CM devised the concept of the report. RF conducted the systemic searching of appropriate literature and was responsible for the drafting of the original piece of work alongside CM. All authors contributed to the article and approved the submitted version.

## Funding

RF is funded by a Department for Education (DfE) NI scholarship to support this work as part of his PhD programme.

## Conflict of interest

The authors declare that the research was conducted in the absence of any commercial or financial relationships that could be construed as a potential conflict of interest.

## Publisher’s note

All claims expressed in this article are solely those of the authors and do not necessarily represent those of their affiliated organizations, or those of the publisher, the editors and the reviewers. Any product that may be evaluated in this article, or claim that may be made by its manufacturer, is not guaranteed or endorsed by the publisher.
